# The Gold Preparations in Some Skin Diseases and Syphilis

**Published:** 1895-04

**Authors:** A. H. Ohmann-Dumesnil

**Affiliations:** Professor of Dermatology and Syphilology in the Marion-Sims College of Medicine, St. Louis; Consulting Dermatologist to the St. Louis City and Female Hospitals; Dermatologist to the Alexian Brothers Hospital, Pius Hospital, Rebekah Hospital, Etc.


### THE GOLD PREPARATIONS IN SOME SKIN DISEASES AND SYPHILIS.

By A. H. OHMANN-DUMESNIL, A. M., M. D.,

Professor of Dermatology and Syphilology in the Marion-Sims College of Medicine, St. Louis; Consulting Dermatologist to the St. Louis City and Female Hospitals; Dermatologist to the Alexian Brothers Hospital, Pius Hospital, Rebekah Hospital, Etc.

THERE are so many preparations of a novel nature, or which are merely revivals of old ones in a new form, being daily offered to the medical practitioner, that he is often at a loss whether to use any of them. Not content with the inherent qualities of these preparations, their promoters either vaunt them as universal panaceas or construct the reading matter so clumsily that one naturally inclined to test the efficacy of the drugs calmly puts them aside until such evidence is forthcoming as will prove convincing and be clearly set forth. It is for this reason that clinical experience is so valuable when based upon careful observation and a knowledge of the conditions present.

Our knowledge of the therapeutical action of gold has, up to within a few years, been based upon the hypothetical dicta of the alchemists. Gradually the matter was taken up again, at first by the Arabian physicians and afterward in Europe. Once more it fell into disuse and was rescued from oblivion by Hahnemann, who introduced it in his pharmacopeia. However, this did not give it much of an impulse and it is only of late years that this metal has undergone any serious investigation concerning its therapeutical properties. Among American investigators Bartholow, Heneage Gibbs, and Shurley are the most prominent. Dr. Shtcherbok has made thorough investigations also.

The most active salt of gold is the bromide, and it is particularly so upon the nervous structures, but small doses being necessary to produce effects. The action of gold is essentially that of an alterative. It has no cumulative effect; but, when toxic doses are administered, mental excitement, amounting to delirium at times, manifests itself. A prominent symptom of its excessive action is an excessive flow of saliva, the so-called *aurism.* Remembering this in connection with the fact that very small doses produce the effects of the remedy, more especially in the form of the bromide, some care should be exercised in its administration.

Among the therapeutic effects of gold may be noted the fact that it is tonic, more especially to the nervous system, and this accounts for the fact that it is an aphrodisiac of no mean power. It was highly esteemed many years ago as an antisyphilitic and recent experience confirms this view, more especially in the later forms of the disease.

The cutaneous troubles in which I have had occasion to employ the gold preparations to any extent are limited. In acne and eczema of a subacute or chronic character I have found arsenauro an invaluable adjuvant. On the other hand, in chronic eczema and in the later manifestations of syphilis, mercauro has proved itself almost a specific; so much so that its administration was always attended by marked improvement, which ceased so soon as it was discontinued. This it was which attracted my attention to the gold preparations, and in investigating their therapeutical properties I have been impressed by the fact that the most active as well as most efficient salt of gold is the bromide. It not only acts powerfully when administered alone, but seems to increase the therapeutic effects of arsenic and of mercury and for that reason much smaller doses of these agents may be given, better results obtained and, at the same time, security from toxic effects will be secured. These are the qualities which recommend the preparations mentioned above, which are true chemical combinations and not empirical mixtures.

It may not be inappropriate to mention a few cases from practice illustrative of the good effects of gold preparations in diseases of the skin and in syphilis :

Case I.—Miss F. B., a dark blonde of seventeen years, has been suffering from a marked pustular acne for two years. She is very nervous in disposition. Vlemingkx's lotion ordered applied at night, and resorcin ointment every morning. In addition, the pustules were emptied thoroughly every day. Some little improvement showed itself, but it was not stable. A "nervous" attack would cause the eruption to manifest itself in a marked relapse. After two months of this treatment, with variations of the external measures, she was placed on ten drops of arsenauro three times a day. In one week the eruption had disappeared, and now, after a lapse of two months, no lesions have appeared.

Case II.—Miss B. R., a blonde of eighteen years, states that she has been suffering from a papular acne, with comedones, since she was thirteen years of age. Free incisions of the papules, together with a sulphur ointment locally, failed to procure any decided effects until arsenauro was used. Her face is now perfectly smooth and clear and downy.

Case III.—Miss M. P. was suddenly attacked by an intense pruritus, accompanied by a mild form of ichthyosis. The ordinary antipruritic solutions did not allay the itching. The peculiar form of pruritus, together with the ichthyotic condition (not congenital), pointed to a central origin of nervous nature. Added to the antipruritic solution was arsenauro, administered in ten-drop doses before each meal. Improvement set in almost immediately, and at the end of three weeks the patient was cured. This occurred despite the fact that the trouble had lasted about a month before I treated her, and despite all the local treatment which she had been given.

Case IV.—C. R., a man of about sixty years, has suffered from general pruritus for several years. At the time he applied for treatment he was emaciated and haggard from loss of sleep due to the obstinate itching from which he suffered. He could only rest after complete exhaustion, and even then sleep was not only not enjoyed, but lasted for the briefest periods only. He presented many of the symptoms of neurasthenia. An antipruritic lotion, to be applied three times daily, and arsenauro, in fifteen-drop doses before meals, have markedly ameliorated his condition. He bids fair to make a complete recovery in a short time.

Case V.—Miss M. Z., a girl of twenty-four years, has been troubled with a rosacea involving almost the entire face for a period of four years. She is of a markedly neurotic temperament, and her skin will become congested visibly if she gets excited in her conversation. The best reducing agents used externally have had but little influence upon the cutaneous affection. Her stomach was not in order, the trouble being, apparently, so-called nervous dyspepsia. Bearing in mind the close relationships between rosacea and gastric disorders, she was ordered five-drop doses of arsenauro before meals in addition to local applications. Marked amelioration of the gastric and dermal symptoms appeared, and now she is practically cured.

Case VI.—Miss H. L., a strong, stout girl of seventeen years, has suffered for a long time from urticaria. A close examination of her case shows that she is intensely susceptible to nervous perturbations. She complained of slight gastric crises at times, which disappeared spontaneously, but were always accompanied by a marked urticarial eruption involving the entire integument, including the scalp. While an antipruritic relieved the condition temporarily, internal measures failed to procure relief until fifteen-drop doses of arsenauro were given and diminished gradually to ten drops three times a day, until the condition was relieved. This relief has now lasted for four months with no indications that the disease will recur.

Case VII.—Mrs. B. H., an old lady of fifty-seven years, had been troubled with a marked case of eczema for a number of years. Her chest, back, arms and thighs, as well as abdomen and breasts, had been the seat of a most intensely itching papular eczema for years. Her forearms and legs were affected with the squamous form of the same disease, the folds of the elbows and the popliteal spaces presenting marked fissures. Constipation which existed was relieved by the acid aperient mixture, and an antipruritic lotion followed by a menthol ointment partially relieved the patient. After a time the condition remained *in statu quo.* As no improvement would appear, she was placed on ten-drop doses of mercauro three times a day, and the dose increased until she took twenty drops at a dose. Improvement was noticeable to such a degree that in one month no traces of the eczema appeared. She was continued on the remedy for two weeks longer, and has continued well ever since, a period of about four months.

Case VIII.—Mr. C. H., an old gentleman of eighty-two years, was troubled with marked eczema of a squamous nature localized upon the backs of his hands. Being placed upon the same treatment as Case VII., much more rapid results were observed. He has had numerous relapses, however, due to the fact that he will handle mortar and similar irritating substances, including frequent washing of the hands. A strict adherence to injunctions, however, is always followed by a rapid return to the normal. Of course, water externally is always prohibited in these cases of eczema.

Case IV.—Mr. A. F., a young man of thirty-two years, applied for treatment for undefined pains in the head and swellings of the forefinger and thumb of the right hand, on the left ramus of the jaw, and over the right clavicle. He had contracted syphilis some five years before. He was given mercauro in fifteen-drop doses, to be increased five drops every week until thirty drops were taken thrice daily, or until vertigo declared itself, when the remedy was to be discontinued for a time. Marked effects for the better declared themselves long before the thirty-drop doses were taken. These latter had to be discontinued after a week on account of the vertigo which declared itself. The swellings, however, had gone down, the backache and headache had disappeared, and the patient slept quietly and was refreshed, something he had not known for a year previously. He resumed the treatment after a rest of two weeks and feels strong and buoyant instead of weak and melancholic.

Case X.—Mr. O. W., a married man of forty-three years, contracted syphilis about two years and a half ago. The condition remained unrecognized for about six months. He then applied to me for treatment. His symptoms disappeared rapidly under active mercurial treatment, followed by the mixed. I lost sight of him for about a year, when he applied for the relief of a bursa of the left knee which had been cut open by a surgeon. It returned, and he was advised an elastic knee cap and placed upon mercauro in twenty-drop doses three times a day. The bursa gradually reduced, and the patient, deeming the elastic bandage sufficient, discarded the medicine. The effusion began increasing and he quickly resumed the mercauro, and I had the satisfaction of observing the effusion entirely disappear. This patient indulged in increased doses which brought on vertigo. This symptom disappeared, however, upon resuming the original quantity. So far as any other syphilitic symptoms are concerned, they are not evident, and the patient feels both cheerful and contented.

These cases have been roughly outlined so as not to weary the reader. They are what might be called sample cases from a large number of similar ones which have terminated favorably under the influence of the gold preparations mentioned. One feature which has been particularly noticed in connection with mercauro is its marked aphrodisiac properties. While only male patients have mentioned this, no doubt the female ones experienced similar sensations or exhilaration. This latter has been alluded to by a number of patients of both sexes. There is no doubt in my mind that the bromide of gold is the most efficient salt of the metal, and it appears to exercise a two-fold effect therapeutically—viz., it increases the action of the arsenic and mercury with which it is combined, and at the same time it seems to prevent the manifestation of toxic symptoms. It is itself very efficient, if we are to believe competent authority, which states positively that bromide of gold is thirty times as efficient as the other bromides.

So far as the preparations mentioned are concerned, they are efficient and rapid in action and the manifest effects of the gold are evident. An exact dosage by means of measuring the drops is attained and ease of administration is secured; no disturbance of the stomach resulting from their ingestion. The vertigo which is experienced disappears as soon as the dose is diminished. I have had no occasion to observe aurism up to the present. In fact, I have seen none but the good effects of these gold preparations. One point, however, must always be borne in mind. The indications presented must be such as demand gold. Some of the older writers maintained that gold was the remedy for syphilis, whereas it is only in the later and deeper manifestations that its good effects are shown. Furthermore, gold and its preparations will not have good effects in all skin diseases, but will prove a most valuable adjuvant in such as have a distinct neurotic base as an etiological or complicating factor.

It is the hope of the writer that the few clinical notes jotted down above may serve as a stimulus to further inquiry into the therapeutical worth and more extended application of gold and its salts, as it is a matter of interest and possibly of the greatest importance, more especially in the treatment of many chronic affections of viscera and organs.—*New York Medical Journal.*

---

New Observations in Gonorrhea.—At the recent meeting of the German dermatological association, considerable time was devoted to the discussion of the etiology of gonorrhea, and among the interesting points brought out, an observation by Wertheim is deserving of especial attention. This careful investigator has found that gonococci obtained from the secretions of chronic gonorrhea can be cultivated so as to acquire a high degree of virulence, and when inoculated in the urethra of the patient from whom they were derived will give rise to an intense gonorrheal inflammation. It has been quite frequently observed that patients suffering from latent gonorrhea at the time of marriage have infected their wives and at a later period acquired from them in return an acute urethral inflammation. Wertheim's experiments are, therefore, of importance in affording a rational and scientific explanation of this clinical observation.—*International Journal of Surgery.*

---

Two Easy and Delicate Tests for Albumin in Urine.—Dr. C. Fouchlos (*Progrés Medical*) recommends two new tests for albumin in urine, for which he claims utmost delicacy and absence of any possible fallacy.

(1) Add to the suspected urine a few drops of a 1 per cent. solution of corrosive sublimate; in case of turbidity, add some drops of acetic acid. If the turbidity persists it is due to the presence of albumin.

(2) Take 100 cc. of a 10 per cent. solution of sulpho-cyanide of potassium, and mix with it 20 cc. of acetic acid. Add a few drops of this mixture to the urine. If albumin is present in small quantities, an immediate turbidity will ensue; if in larger quantities, a heavy white precipitate will appear.—*Med. and Surg. Reporter.*

